# Publisher Correction: Operando monitoring the lithium spatial distribution of lithium metal anodes

**DOI:** 10.1038/s41467-018-05212-6

**Published:** 2018-07-19

**Authors:** Shasha Lv, Tomas Verhallen, Alexandros Vasileiadis, Frans Ooms, Yaolin Xu, Zhaolong Li, Zhengcao Li, Marnix Wagemaker

**Affiliations:** 1 Department of Radiation Science and Technology, Delft University of Technology, Mekelweg 15, Delft 2629 JB, The Netherlands; 2 The State Key Laboratory of New Ceramics and Fine Processing, School of Materials Science and Engineering, Tsinghua University, Beijing 100084, China; 3 Key Laboratory of Advanced Materials (MOE), School of Materials Science and Engineering, Tsinghua University, Beijing 100084, China

Correction to: *Nature Communications*; 10.1038/s41467-018-04394-3; published 01 June 2018

The original HTML version of this Article omitted to list Zhengcao Li as a corresponding author. Correspondingly, the original PDF version of this Article incorrectly stated that ‘Correspondence and requests for materials should be addressed to M.W. (email: m.wagemaker@tudelft.nl)’, instead of the correct ‘Correspondence and requests for materials should be addressed to Z.L. (email: zcli@tsinghua.edu.cn) or to M.W. (email: m.wagemaker@tudelft.nl)’. This has been corrected in both the PDF and HTML versions of the Article.

