# Supplementary material for: Operando monitoring the lithium spatial distribution of lithium metal anodes
Source: Nat Commun. 2018 Jun 1;9:2152. doi: 10.1038/s41467-018-04394-3 (PMC5984624; doi:10.1038/s41467-018-04394-3)
Supplement: Supplementary file 1 — Supplementary Information [file 41467_2018_4394_MOESM1_ESM.pdf]

Supplementary Information

Operando monitoring the Lithium spatial distribution of Lithium metal anodes

Lv et al.

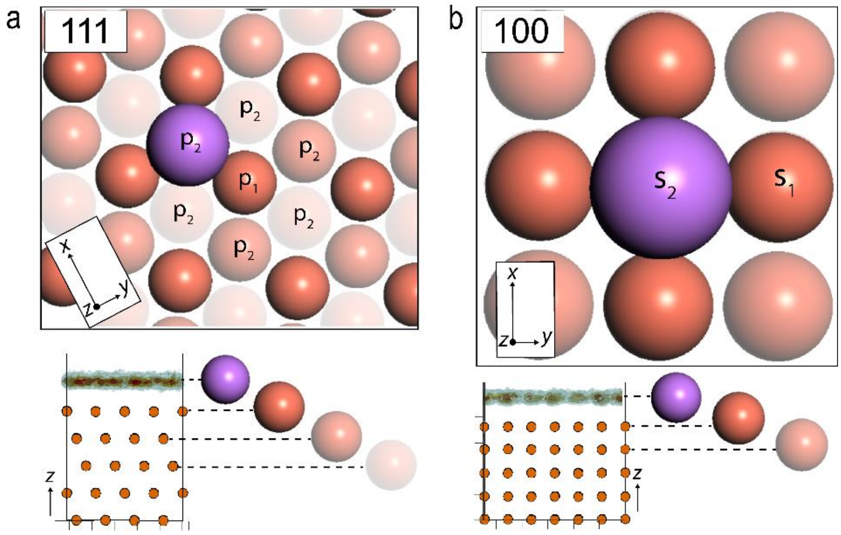

Supplementary Figure 1: Li on copper surfaces viewed perpendicular and parallel to the surface. **a** The notations p1 and p2 represent the coplanar 2D, Cu(111) surface positions at a distance of ~2.1 Å above the copper layers. The p2 position is directly projecting the second or third copper layer of the Cu(111) surface while the p1 position is diametrically projecting the first Cu layer of the Cu(111) surface. **b** The notations s1 and s2 represent the coplanar 2D, Cu(100) surface positions we defined at a distance of ~2.1 Å above the copper layers. The s2 and s1 positions are directly projecting the second and first copper layer of the Cu(111) surface respectively. When Li was initialised on the p1 "unfavourable" position it relaxes towards the p2 position during the energy optimization. The same trend was observed for the Cu(100) surface.

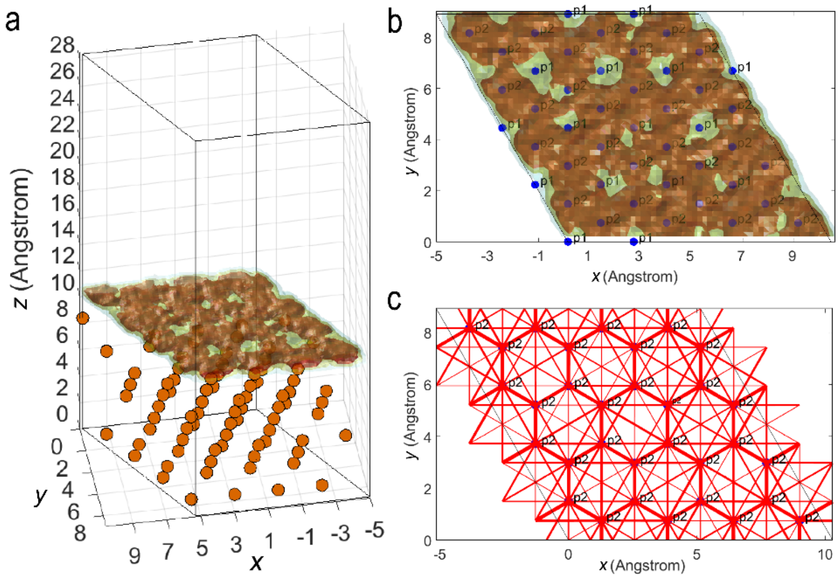

Supplementary Figure 2: Li-ion mobility on the Cu (111) surface by MD simulation. **a** Integrated Li density of 4 diffusing Li atoms on the (111) copper surface for a 94 ps MD simulation at 600 K. **b** Perpendicular to the surface view of the Li density and the surface p1 and p2 positions (blue spheres). **c** Detected transitions presented as red lines, resulted from sampling the surface with the p2 positions. The thickness of the lines scales with the number of transitions observed.

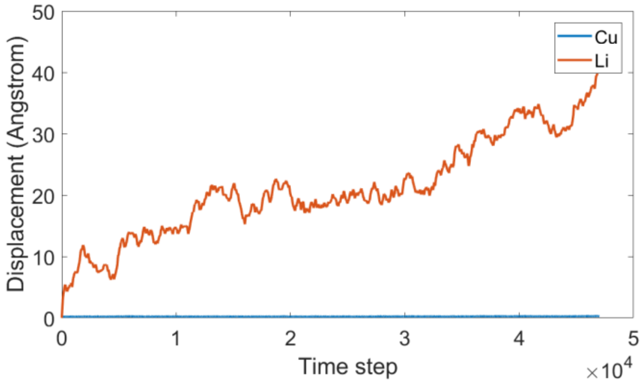

Supplementary Figure 3: Total displacement of 4 Li on the Cu(111) surface throughout the 94 ps of the MD simulation at 600K.

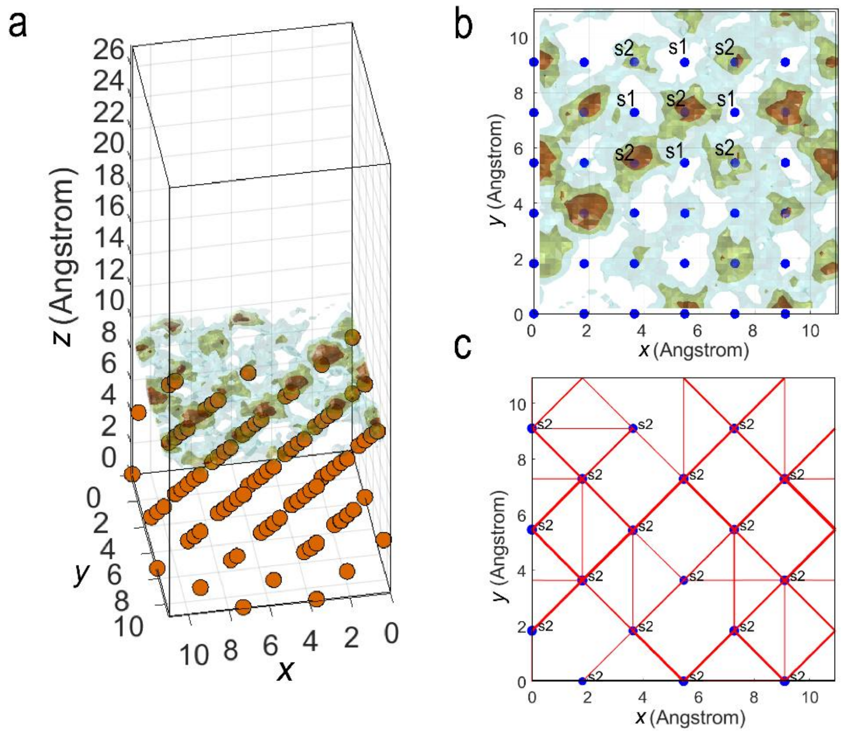

Supplementary Figure 4: Li-ion mobility on the Cu (111) surface by MD simulation. **a** Integrated Li density of 1 diffusing Li atoms on the (100) copper surface for a 105 ps MD simulation at 600 K. **b** Perpendicular to the surface view of the Li density and the surface s1 and s2 positions (blue spheres). **c** Detected transitions presented as red lines, resulted from sampling the surface with the s2 positions. The thickness of the lines scales with the number of transitions observed.

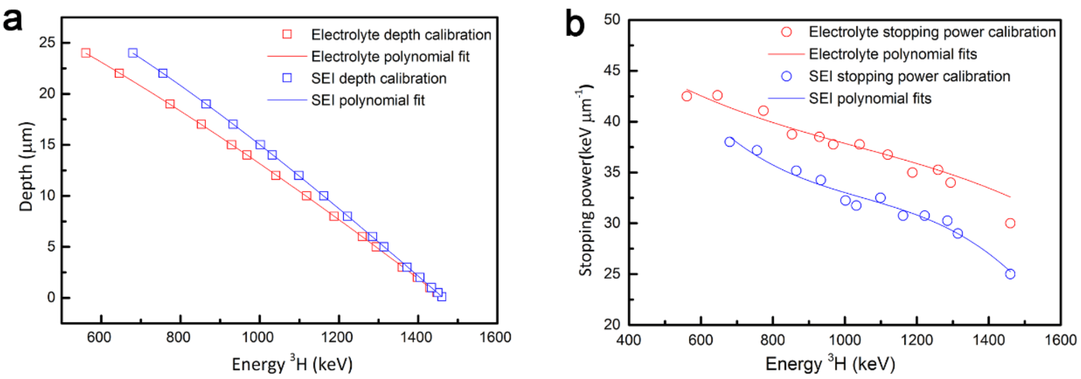

Supplementary Figure 5: Comparison of the NDP calibration for the electrolyte and SEI. **a** Depth calibration and **b** stopping power for the electrolyte (1 M LiPF6 in 1:1 EC:DMC) and a typical SEI density (for Li-metal in combination with 1 M LiPF6 in 1:1 EC:DMC) based on[1].

Supplementary Table 1: Adsorption Energies of Li on copper surfaces (eV per Li)

$E_{ad,Li}(111)$	–0.71
$E_{ad,Li}(100)$	–0.58

Supplementary Methods

DFT relaxation of bulk face-centered cubic Cu lattice resulted in a lattice parameter of 3.627 Å, which compares extremely well, << 1 %, with the experimentally determined values[2,3] of 3.614 Å and 3.595 Å as well as with values (3.649 Å, 3.571 Å)[4,5] obtained from similar computational studies. To evaluate the possibility of Li insertion in the bulk lattice of copper, we placed Li in a 2x2x2 copper supercell ($Li_{0.03}Cu$) and performed DFT calculations. Both the empty 4b and 8c (according to Wyckoff notation) interstitial positions of the Fm-3m crystal lattice were investigated. The average insertion voltage (V) can be determined as by equation (1)[6]:

$$V = -\frac{E_{Li_x bulk} - xE_{Li} - E_{bulk}}{xe} \qquad (1)$$

where $E_{Li_x bulk}$ is the total energy of the Li-copper configuration at a certain concentration x, E_{Li} is the Li energy and E_{bulk} is the total energy of the empty copper configuration. Highly negative potentials of -2.43 and -2.99 V for Li insertion in the 4b and 8c site respectively were determined, indicating that Li insertion into the bulk copper is unlikely.

The optimized bulk Cu lattice constant was used for the building the Cu(111) and Cu(100) surfaces in 10.3 x 10.3 x 28.4 $Å^3$ ($\angle xy = 120°$) and 10.9 x 10.9 x 27.3 $Å^3$ $\angle xy = 90°$) supercell configurations, containing in total 80 and 90 copper atoms respectively. Both the Cu(111) and Cu(100) supercells include 5 copper layers and a large vacuum slab of 20 Å to avoid interactions perpendicular to the surface direction[7]. Surface energies were obtained with a 600 eV cut-off energy and a, reduced to the long direction, 11 x 11 x 1 *k*-point mesh. The thermodynamic stability of the copper surfaces can be evaluated by computing the surface energy γ which is given by equation (2)[4,7]:

$$\gamma = \frac{E_{slab,r} - nE_{bulk}}{A} - \frac{E_{slab,u} - nE_{bulk}}{2A} \qquad (2)$$

where $E_{slab,r}$ is the energy of the relaxed slab, E_{bulk} the energy of the primitive cell, $E_{slab,u}$ is the energy of the unrelaxed slab, n is the number of atoms in the slab and A is the surface area of one side of the slab. The Cu(111) and Cu(100) surface energies were determined resulting in γ_{111} = 1.294 Jm^{-2} (0.081 eV $Å^{-2}$) and γ_{100} = 1.424 Jm^{-2} (0.089 eV $Å^{-2}$), which are in excellent agreement with previous studies utilizing the PBE exchange correlation functional[5,8]. Having a lower surface energy, the Cu(111) surface is determined to be more thermodynamically stable than the Cu(100) surface, in line with the dominant surface observed[5,7-10] that consistently report a $\gamma_{r111} < \gamma_{r100} < \gamma_{r110}$ relative stability of the surfaces. The two most stable surfaces, Cu(111) and Cu(100), are shown to dominate the Wulff construction[5] whereas sometimes small contributions of the third most stable surface, Cu(110), are included[4].

In order to investigate the lowest energy positions of Li at the copper surfaces Density Functional Theory (DFT) relaxation calculations were performed. For the Cu(111) surface, Li stabilizes at a vertical distance of 2.1 Å from the outer copper layer, a distance that is equivalent to the one between the Cu(111) copper layers. **Supplementary Figure 1** Li is coordinated by the three nearest copper atoms of the first outer layer having Cu-Li interatomic distances of 2.585 Å which is comparable to the respective Cu-Cu distances of the inner layers (2.57 Å). Energy minimization of Li placement on top of the Cu(100) resulted in Li obtaining the lowest energy when directly projecting the second in depth Cu layer (defined as s2 position), maximizing its distance from the first copper layer (defined as s1 position), as shown in **Supplementary Figure 1**. The adsorption energy is defined as the energy difference between the slab with Li adsorbed on the surface ($E_{slab,r+Li}$) and the energy sum of the relaxed empty slab ($E_{slab,r}$) and the Li (E_{Li}).

$$E_{ads,Li} = E_{slab,r+Li_{ads}} - (E_{slab,r} + E_{Li}) \quad (3)$$

The obtained adsorption energies are presented in **Supplementary Table 1**. Negative adsorption energies, according to the formulation presented herein, indicate that Li adsorption on the copper surfaces is thermodynamically stable.

Supplementary References

1 Vatamanu, J., Borodin, O. & Smith, G. D. Molecular Dynamics Simulation Studies of the Structure of a Mixed Carbonate/LiPF6 Electrolyte near Graphite Surface as a Function of Electrode Potential. *J. Phys. Chem. C* **116**, 1114–1121, (2012).

2 Strauman, M. E. & Shah, J. S. Lattice Parameters, Densities, Expansion Coefficients and Perfection of Structure of Cu and of Cu-In a Phase. *Acta Crystall.* **A 25**, 676–682 (1969).

3 Csonka, G. I. *et al.* Assessing the performance of recent density functionals for bulk solids. *Phys. Rev. B* **79**, 155107 (2009).

4 Tafreshi, S. S., Roldan, A., Dzade, N. Y. & de Leeuw, N. H. Adsorption of hydrazine on the perfect and defective copper (111) surface: A dispersion-corrected DFT study. *Surf. Sci.* **622**, 1-8, (2014).

5 Fishman, M., Zhuang, H. L. L., Mathew, K., Dirschka, W. & Hennig, R. G. Accuracy of exchange-correlation functionals and effect of solvation on the surface energy of copper. *Phys. Rev. B* **87**, 245402 (2013).

6 Aydinol, M. K., Kohan, A. F., Ceder, G., Cho, K. & Joannopoulos, J. Ab initio study of lithium intercalation in metal oxides and metal dichalcogenides. *Phys. Rev. B* **56**, 1354-1365, (1997).

7 Tafreshi, S. S., Roldan, A. & de Leeuw, N. H. Density Functional Theory Study of the Adsorption of Hydrazine on the Perfect and Defective Copper (100), (110), and (111) Surfaces. *J. Phys. Chem. C* **118**, 26103–26114, (2014).

8 He, L., Liu, Y. W., Tong, W. J., Lin, J. G. & Wang, X. F. Surface Energy Engineering of Cu surface by strain: First-Principles Calculations. *Surf. Rev. Lett.* **20**, 13500546 (2013).

9 Lee, B. J., Shim, J. H. & Baskes, M. I. Semiempirical atomic potentials for the fcc metals Cu, Ag, Au, Ni, Pd, Pt, Al, and Pb based on first and second nearest-neighbor modified embedded atom method. *Phys. Rev. B* **68**, 144112 (2003).

10 Wen, Y. N. & Zhang, H. M. Surface energy calculation of the fcc metals by using the MAEAM. *Solid State Commun.* **144**, 163–167, (2007).
